# Supplementary material for: Proteogenomic Profiling of Treatment-Naïve Metastatic Malignant Melanoma
Source: Cancers (Basel). 2025 Feb 27;17(5):832. doi: 10.3390/cancers17050832 (PMC11899103; doi:10.3390/cancers17050832)
Supplement: Supplementary file 1 [file cancers-17-00832-s001.zip › Supplementary_Information.pdf]

Proteogenomic profiling of treatment naïve metastatic malignant melanoma

Magdalena Kuras [1,2,†], Lazaro Hiram Betancourt [1,3,*,†], Runyu Hong [4,5†], Leticia Szadai [6], Jimmy Rodriguez [7], Peter Horvatovich [2,8], Indira Pla [2], Jonatan Eriksson [2], Beáta Szeitz [9], Bartłomiej Deszcz [10], Charlotte Welinder [3], Yutaka Sugihara [2], Henrik Ekedahl [3,11], Bo Baldetorp [3], Christian Ingvar [11,12], Lotta Lundgren [3,11], Henrik Lindberg [2], Henriett Oskolas [3], Zsolt Horvath [2], Melinda Rezeli [2], Jeovanis Gil [1], Roger Appelqvist [2], Lajos V. Kemény [13,14,15,16], Johan Malm [1], Aniel Sanchez [1], Attila Marcell Szasz [17], Krzysztof Pawłowski [1,10,18], Elisabet Wieslander [1,*], David Fenyö [4,5,‡], Istvan Balazs Nemeth [6,‡] and György Marko-Varga [2,19,20,‡]

1 Department of Translational Medicine, Lund University, Skåne University Hospital Malmö, 214 28 Malmö, Sweden; magdalena.kuras@bme.lth.se (M.K.); jeovanis.gil_valdes@med.lu.se (J.G.); johan.malm@med.lu.se (J.M.); aniel.sanchez@med.lu.se (A.S.); krzysztof.pawlowski@utsouthwestern.edu (K.P.)

2 Department of Biomedical Engineering, Lund University, 221 00 Lund, Sweden; p.l.horvatovich@rug.nl (P.H.); indira.pla_parada@bme.lth.se (I.P.); jonatan.eriksson@axis.com (J.E.); yutaka.sugihara@med.lu.se (Y.S.); henrik.lindberg@med.lu.se (H.L.); hzsmisi@gmail.com (Z.H.); melinda.rezeli@bme.lth.se (M.R.); roger.appelqvist@bme.lth.se (R.A.); gyorgy.marko-varga@bme.lth.se (G.M-V.)

3 Department of Clinical Sciences Lund, Division of Oncology, Lund University, 221 00 Lund, Sweden; charlotte.welinder@med.lu.se (C.W.); henrik.ekedahl@med.lu.se (H.E.); bo.baldetorp@med.lu.se (B.B.); lotta.lundgren@med.lu.se (L.L.); henriett.kovacsne_oskolas@med.lu.se (H.O.)

4 Institute for Systems Genetics, NYU Grossman School of Medicine, New York, NY 10016, USA; rh2740@nyu.edu (R.H.); david@fenyolab.org (D.F.)

5 Department of Biochemistry and Molecular Pharmacology, NYU Grossman School of Medicine, New York, NY 10016, USA

6 Department of Dermatology and Allergology, University of Szeged, 6720 Szeged, Hungary; szadai.leticia@med.u-szeged.hu (L.S.); nemeth.istvan.balazs@med.u-szeged.hu (I.B.N.)

7 Department of Biochemistry and Biophysics, Karolinska Institute, 171 77 Stockholm, Sweden; jimmy.rodriguez@thermofisher.com

8 Department of Analytical Biochemistry, Faculty of Science and Engineering, University of Groningen, 9712 CP Groningen, The Netherlands

9 Division of Oncology, Department of Internal Medicine and Oncology, Semmelweis University, Budapest, Hungary Institute for Systems Genetics, NYU Grossman School of Medicine, New York, NY 10016, USA; beata.szeitz@nyulangone.org

10 Department of Biochemistry and Microbiology, Warsaw University of Life Sciences, 02-787 Warsaw, Poland; bartek_deszcz@wp.pl
11 SUS University Hospital Lund, 222 42 Lund, Sweden
12 Department of Surgery, Clinical Sciences, Lund University, SUS, 221 00 Lund, Sweden, 221 00 Lund, Sweden; christian.ingvar@med.lu.se
13 HCEMM-SU Translational Dermatology Research Group, Semmelweis University, 1085 Budapest, Hungary; kemeny.lajos@semmelweis.hu
14 Department of Dermatology, Venereology and Dermatooncology, Faculty of Medicine, Semmelweis University, 1085 Budapest, Hungary
15 Department of Physiology, Faculty of Medicine, Semmelweis University, 1085 Budapest, Hungary
16 MTA-SE Lendület "Momentum" Dermatology Research Group, Hungarian Academy of Sciences and Semmelweis University, 1085 Budapest, Hungary
17 Department of Bioinformatics, Semmelweis University, 1085 Budapest, Hungary; szasz.attila_marcell@med.semmelweis-univ.hu
18 Department of Molecular Biology, University of Texas Southwestern Medical Center, Dallas, TX 75390, USA
19 Chemical Genomics Global Research Lab, Department of Biotechnology, College of Life Science and Biotechnology, Yonsei University, Seoul 03722, Republic of Korea
20 1st Department of Surgery, Tokyo Medical University, Tokyo 160-8402, Japan

* Correspondence: lazaro_hiram.betancourt_nunez@med.lu.se (L.H.B.); elisabet.wieslander@med.lu.se (E.W.)
† These authors contributed equally to this work.
‡ These authors also contributed equally to this work as senior authors.

The authors declare no potential conflicts of interest

Supplementary Materials and Methods

Sample acquisition

Discovery cohort

The tissue specimens in the discovery cohort were snap-frozen or put on dry ice within 30 minutes of collection; most samples were frozen within 15 minutes upon surgery, with a small amount of isopentane in liquid nitrogen. Multiple pieces were collected from most of the tumor specimens. The samples were then stored at -80 ℃ in the Melanoma biobank, BioMEL, Region Skåne, Sweden.

Histopathological analysis

Discovery cohort

Stepwise sectioning of the tissues was performed, and on average, three sections were evaluated for each tumor. Frozen tissue sections were placed on glass slides, stained with hematoxylin and eosin, and then placed in an automated slide scanner system (Zeiss Mirax). The tissue content was then evaluated in terms of tumor cells, necrosis, connective tissue, and adjacent background tissue, and features that could be further captured based on morphology were considered. Necrosis did not affect the quality of the data. Only 10 out of 142 samples of the cohort have necrosis >20%, and most of them (6) displayed high tumor content (>60%). Therefore, no samples were excluded based on necrosis.

For deeper evaluation, we assessed the properties of the tumor cells (primary pattern, size) and the infiltration of lymphocytes in the tumor mass (an immunoscore was given representing tumor-infiltrating lymphocytes both in the dimension of intensity and extent). The assessment was performed by a board-certified pathologist. To improve the comparability of studies, the methods were adjusted to the at-that-time existing protocol of The Cancer Genome Atlas Network, and their analysis of malignant melanoma (TCGA, Cell 2015). This approach was followed as briefly described below. The following pathologic parameters were scored for each case: lymphocyte distribution (0-3, 0 = no lymphocytes within the tissue, 1 = lymphocytes present involving <25% of the tissue cross-sectional area, 2 = lymphocytes present in 25 to 50% of the tissue, 3 = lymphocytes present in >50% of tissue); lymphocyte density (0-3; 0 = absent, 1 = mild, 2 = moderate, 3 = severe). The pathologist was provided with written definitions for

each parameter and an illustrated guide. A lymphocyte score defined as the sum of the lymphocyte distribution and density scores (0-6) was also calculated for each case.

Quality control of the MS analysis

Quality control measurements were introduced to assess the performance of LC-MS/MS systems. A protein digest from HeLa cells (Pierce HeLa Protein Digest Standard, Thermo Fisher Scientific) mixed with a standard peptide mixture (Pierce Peptide Retention Time Calibration Mixture) was used as a QC sample and measured every tenth LC-MS/MS analysis. This allowed monitoring of the peak width, retention time, base peak intensity, number of MS/MS, Peptide-Spectrum matches (PSMs), and number of peptides and proteins identified.

Evaluation of the TMT global proteomic data

The TMT-based proteomic analyses of 142 metastatic malignant melanoma samples resulted in the identification of 12,695 proteins (11,468 genes) with an average of 10,705 proteins identified per sample. The data displayed 15.5% missing values for the protein abundances and 8,124 proteins were present across all samples. Long-term reproducibility of the digestion workflow was previously shown [141]. The reliability of the TMT workflow was evaluated by repeating the entire experiment of batch one. Although factors such as sample aging and change of RP-high pH fractionation column and MS instrument influenced the analysis, the overall agreement and correlation of protein abundances between the experiments were good (Figure S1C). In addition, good longitudinal performance across the 15 batches was demonstrated by the rather constant sequence coverage (Figure S1D).

Principal component analysis using 8,124 proteins quantified in all the 142 melanoma samples could separate between high- (>70%) and low-containing (<30%) tumor samples based on protein abundance (Figure S1E). No batch effects were observed for the global proteomic or phosphoproteomic data (Figures S1E and S1F). mRNA and protein abundances showed a strong positive correlation (median 0.408), and 84% showed a significant correlation ($p<0.05$) for the 6,101 overlapping genes across 104 patient samples (Figure S1G). The average correlation was in the range of previously reported mRNA-protein correlations from CPTAC proteogenomic studies.

Evaluation of the phosphoproteomic data

The sample preparation workflow was previously assessed for its reliability using malignant melanoma tissue samples [23]. Principal component analysis, using 1,267 phosphosites commonly quantified among 118 patient samples showed similar separation as for the global

proteomic dataset, separating the high- (>70%) and low-containing (<30%) tumor samples based on phosphosite abundance (Figure S1F). Protein and phosphoprotein abundances showed a strong positive correlation (median 0.506), and a 94% significant correlation ($p<0.05$) for the 809 overlapping proteins across 94 patient samples (Figure S1H).

Statistical analysis of omics data

Sample exclusion

For consensus clustering and subtype analysis, samples with >30% tumor content (n=118) were included, while the analysis of phenotype-switching markers was restricted to samples with >70% tumor content (n=83) (Figure S1J). Independent component analysis (ICA) was performed on samples with >30% tumor content, and BRAF V600 mutation analysis was conducted on n=49 samples. Tumor microenvironment (TME) analysis was limited to samples with <50% tumor content (n=29). Survival analyses were carried out on samples with >30% tumor content, including outlier and Cox regression. Sample MM-SEG-0113 was excluded from all analyses due to technical issues.

Consensus clustering analyses

Unsupervised-consensus hierarchical clustering analysis was performed using the 3,000 proteins with the most variable expression levels (coefficient of variation > 0.36) using the Perseus software (v 1.6.14.0) [25]. The clustering algorithm used k-means, Pearson correlation distance, and average linkage. Five subgroups were identified as proteomic subtypes by visually examining the hierarchical tree.

Subtypes and feature correlation

Correlations between subtypes and clinical and histopathological features and transcriptomic subtypes were performed using Fisher's exact test. Kruskal-Wallis tests were performed on the histological parameters, including the contents of tumor cells, adjacent lymph nodes, tumor-associated connective tissue, and necrosis.

Differential omics analysis

To identify proteins, phosphosites, and transcripts differentially expressed across the subtypes, one-way ANOVA was performed using the TMT-based global proteomic, phosphoproteomic, and transcriptomic data, respectively. At least 30% of valid values were required for all datasets and the p-values were adjusted using the permutation-based FDR method.

Independent component analysis

Pre-processed and normalized proteomic, transcriptomic, and phosphoproteomic data were dimensionally reduced by independent component analysis (ICA) separately [142]. To ensure the quality of the ICA, we only included omics data of samples with tumor content above 30%. An R-based package, "fastICA", was used for implementation. The ICA was performed 100 times for each omics dataset to make sure that the ICs were consistent. The extracted independent components (ICs) mixing scores of the omics data were then passed through association tests with the joint table of clinical features of patients in our cohort. If the clinical variable is binary, a logistic regression model was built for association tests. Otherwise, a linear regression model was built. The association tests were conducted for all the 100 ICA analyses for each omics dataset and its ICs, and the ICs showing correlations (p-value<0.005) with a clinical feature for at least 30 ICA runs per 100 were picked as significant. For each of these significant ICs, the centroid of IC coefficients was used to rank the omics data. We then used these rankings to conduct Gene Set Enrichment Analysis (GSEA) and significant pathways were found (adjusted p-value<0.01). The GSEA was implemented by an R-based package, "fgsea", and searched against the "Reactome" database. The ICs served as links between clinical and histological features and pathways.

Identification of mortality risk subgroups of BRAF V600 mutated patients

The R package 'InGRiD' 130 (Integrative Genomics Robust iDentification of cancer subgroups) was applied to identify subgroups of patients with different mortality risk rates within our cohort of 49 patients with a BRAF mutation (Table S1A). The package provides a pathway-guided identification of patient subgroups based on protein expression while utilizing patient survival information as the outcome variable. The analysis was supplied with the relative abundances of proteins differentially expressed between tumors with high and low expression of the mutation and the associated pathways. These proteins were extracted from a previous study [68]. As the outcome variable, we considered the patient survival time from sample collection to death or censoring (DSS). All default parameters in 'InGRiD' were kept.

Custom database construction and Single Amino Acid Variant (SAAV) peptide identification

A custom protein sequence database was built by downloading protein mutation data from the Cancer Mutant Proteome Database [143]. This included the skin cutaneous melanoma data from TCGA (369 cases) and 7 melanoma cell lines from the NCI-60 panel. Additional data on

melanoma was retrieved from COSMIC v80 (downloaded 2017-02-15). Protein IDs, mutation or variant positions, and mutated protein sequences were extracted, and a UniProt ID was assigned to the proteins, using a custom script written in Tcl. Using the matched protein sequence, a peptide that carried the mutation site was generated by performing an in silico tryptic digestion of the protein and allowing for one additional missed cleavage at both sides of the mutation site. Redundant mutations were then removed and entries with the same mutated peptide sequence were grouped into one single entry. The resulting database comprised 57,134 entries.

Raw files were processed with Proteome Discoverer 2.3 using the Sequest HT search engine in a two-step search. The first search was performed against the Homo Sapiens Swissprot database (see "TMT11 plex quantification of proteomic data"), and unassigned MS/MS spectra were searched against the above-described in-house built database. Cysteine carbamidomethylation was set as fixed modification while methionine oxidation and TMT11 plex at peptide N-terminus and lysine were set as variable modifications; peptide mass tolerance for the precursor ions and MS/MS spectra were set to 10 ppm and 0.02 Da, respectively. A maximum of two missed cleavage sites were accepted and FDR was set at 1% for identification at the peptide level.

Validation of SAAV search results

SAAV peptides were validated using the SpectrumAI quality control tool available as an R script [144]. A custom R script was used for data cleanup and post-processing. The verified SAAVs pointing at the same mutation position on a protein were merged into one entry. The reason for multiple entries includes missed cleavages as well as complementary peptides pointing at the same mutation. The latter occurred if the amino acid change generated a new trypsin cleavage site leading to a peptide that cannot be predicted from the original canonical sequence.

For peptides assigned to an isoform of the master protein, the mutation positions were corrected to reflect the position in the canonical Uniprot sequence. This was performed by using a customized R script analyzing the UniProtKB isoform sequences (accessed 2019-08-21). Both the corrected and uncorrected position was used for online database searches, to ensure that we would not miss matching results due to the position disparity caused by isoform sequences. Additionally, the Peptide-Spectrum Matches (PSMs) matching wild-type peptides originating from the normal database search were linked to the corresponding SAAVs, which allowed

assessment of the ratio of wild-type and SAAV peptide PSMs. KEGG and GO enrichment analyses were done using the clusterProfiler R package. Signaling pathway members were obtained from KEGG, and UniProt accession IDs were converted to KEGG IDs using the KEGG Mapper [145].

Annotation of validated SAAVs

Merging the results of various database searches and cleaning the data was performed with in-house custom R scripts. First, the SAAV peptides were searched in PeptideAtlas database [146] to determine if the SAAV peptides were observed previously. The search was performed on the webpage https://db.systemsbiology.net/sbeams/cgi/PeptideAtlas/GetPeptides using the "Human 2020-01" Atlas Build, only keeping the canonical and isoform protein accession numbers for which SAAVs were identified in our study. The resulting peptide sequences were downloaded in text format, and a custom R script was used to retrieve exact and partial matches.

Validated coding SNPs and cancer-related mutations were downloaded from the CanProVar database [147]. The UniProt IDs were first converted to Ensembl IDs using the biomaRt (version 2.42.1) R package [148], and then the CanProVar database was used to retrieve the variant's reference SNP ID (rs#) and any cancer-related variation ID of CanProVar. The "Index of Human Polymorphisms and disease mutation" document was downloaded from UniProt (https://www.uniprot.org/docs/humsavar) and was also used to retrieve reference SNP IDs.

Validated SAAVs and the corresponding genes were searched for in The Cancer Gene Census (CGC) [149]. Additionally, we used the bioinformatic predictor FATHHM (functional analysis through hidden Markov models, http://fathmm.biocompute.org.uk/cancer.html, Hum. Mutat., 34:57-65) using the recommended Prediction Threshold of -0.75 to look for cancer-associated variants in our SAAV dataset.

Signature of melanoma-associated SAAVs

Due to the lack of genomic and mutational data on individual tumor samples, a signature of 167 melanoma-associated SAAVs was extracted. The signature was classified into levels 1-3 and included SAAVs with markedly different occurrences in the melanoma samples compared to the healthy tissue of patients.

Data on aggregated allele frequency, named here as Variant Allele frequency (VAf) were accessed using the NCBI Variation Service API as described in https://github.com/ncbi/dbsnp/blob/master/tutorials/Variation%20Services/Jupyter_Notebook/by_rsid.ipynb. For this analysis, a custom Python script was used. Additional resources such as ExAc [150], 1000Genomes [151], and HapMap [152] were used to manually retrieve VAf information when this information was missing. VAf in the European population (AAFs) were manually imputed for BRAF V600E, NRAS Q61K/R, HRAS G13D, and CDKN2A P114L. For BRAF V600E, HRAS G13D, and CDKN2A P114L the global population alternative allele frequency was used. VAfs for NRAS mutations were inferred both from BRAF V600E frequency and melanoma COSMIC data. According to COSMIC, BRAF mutation occurs 2.6471 times more than NRAS mutation (45% and 17% of the melanoma patients have these gene mutations, respectively), as well as NRAS Q61R occurs 1.067 times more frequently than NRAS Q61K (as 784 and 735 patients had Q61R and Q61K mutations, respectively). Based on these ratios, alternative allele frequency in the European population was inferred as AAFBRAF Q61R = 3.1E-06 and AAFNRAS Q61K = 2.9E-06.

Level 1

First, we estimated the frequency of SAAV (SAAVf) in the tumors as:

SAAVf = (nSAAV PSM) / (nSAAV PSM + nwild-type PSM), where nSAAV PSM is the number of PSMs supporting the SAAV peptide, while nwild-type PSM is the number of PSMs supporting the wild-type peptide (Table S5A). The SAAVf displayed a significant correlation (Spearman 0.75, p-value=2.2e-16) with the corresponding Vaf (Figure S5A). Next, an enrichment factor SAAVr was defined as: SAAVr = SAAVf/ VAfE, where VAfE is the alternative allele frequency in the European population. Log10-transformed SAAVr values were subjected to Johnson transformation using Minitab (vs 17) to achieve values following the normal distribution (M = 0.01304, SD = 0.9988). The significance level was set to $\alpha = 0.1$, and values outside of the range [-1.630; 1.656] were considered under- (n=49) or over-represented (n=34) SAAVs (Figure 4B and Table S5A).

Level 2

In total, 22 SAAVs (Table S5A) were defined as: 1) VAfE < 0.3; 2) no detection of the wild-type peptide in our TMT experiment; and 3) evidence of the wt peptides in the Peptide Atlas database (Figure 4C and Table S5A). Thus, they likely constitute over-represented SAAVs in melanoma tumors.

Level 3

In total, 62 SAAVs (Table S5A) for which VAf was not available and could not be estimated (Figure 4C and Table S5A). In eight of the cases, the wt-peptide was not detected despite being identified in proteomic studies reported in the Peptide Atlas database. The absence of VAf data could be seen as an indicator of low occurrence in the population, with a potential association with melanoma.

Discovery of melanoma SAAV-based neoantigens

Peptides-containing SAAVs and their position within respective protein sequences were aligned with a database of a melanoma-associated immunopeptidome [100]. The NetMHCpan-4.1 tool [101] was used to evaluate the MHC I binding of database-matched peptides and SAAV-altered peptide counterparts.

Tumor microenvironment analyses

The adjacent lymph node (LN) and tumor-derived connective tissue (CT) contents separated the cohort into two groups each: the high LN group (HLN) with LN > 27% and the low LN group (LLN) with LN ⩽ 27%, and the high CT (HCT) group with CT > 45.5%, and the low CT group (LCT) with CT ⩽ 45.5%. Connective tissue includes tumor-derived stroma and adipose tissue. The cut-off values were selected by ROC curves based on the ability of these histological features to discriminate between long and short survivals, considering a three-year survival from sample collection (DSS), and below 50% tumor content. Kaplan-Meier survival analysis with log-rank (Mantel-Cox) and Gehan-Breslow-Wilcoxon testing was used for univariate analysis between these groups. P-value < 0.05 was considered statistically significant, and values for patients reported as alive, with an "unknown cause of death" or "dead due to other reasons" were censored. These analyses were performed using Graph Pad Prism version 9.1.1.

Survival biomarker analyses

Two complementary supervised approaches were used to relate omics data to survival. First, Outlier analysis, which treats survival as a binary variable. Second, Cox analysis considers survival as a continuous variable.

Outlier Analysis

Outlier analyses were performed for different variables of interest including survival, BRAF mutation, NRAS mutation, gender, and tumor stage. For the survival-related variables, the dataset was divided into 2 groups and binary variables were created based on whether the patients lived longer than certain cutoff times, or not. The cutoff times used were 6 months, 1 year, 3 years, and 5 years from their sample collection date (DSS). Outlier analyses were conducted to find which proteins were significantly enriched in one group. We used a Python-based package, “BlackSheep”, to implement these analyses on the proteomic, transcriptomic, and phosphoproteomic data separately with a default median and interquartile range (IQR) of 1.5. Significant genes were picked by an FDR cutoff at 5% in the group-wise comparisons. Proteins labeled as outliers in less than 30% of the patient samples in one group were excluded from the group-wise comparisons.

Cox’s proportional hazards survival analysis

In addition, we performed survival analysis using regularized Cox regression in a similar manner as previously published [153]. The samples were randomly split into a training and a test set (80-20 training-test set). Using the training samples, a univariate Cox model was fitted for each feature individually and the 30 features with the lowest univariate p-values were selected and used as input to an elastic-net Cox model (Table S8B). The C-index was computed on the left-out test samples. This procedure was repeated 100 times for each omics dataset. We then considered the features that were selected by the Cox model in at least 50 of the 100 repetitions as significant and investigated these further.

A C-index of 1.0 means that the model "ranks" the samples perfectly, i.e., patients with a higher risk score (hazard score) died earlier than those with lower scores. A C-index of 0.5 is the expected performance of a random model. The C-index parameter is analogous to the AUC parameter (Area Under Curve) used for a binary classifier. The predictive power of our molecular data was moderate, and concordance indices (C-indices) varied between 0.538 and 0.601.

ROC curve analysis

The survival data of the patients were divided at 6 months, 1 year, 3 years, and 5 years into binary variables. Univariate receiver operating characteristic (ROC) curves of each binary

survival variable and each protein expression were constructed by the ‘pROC’ package in R. Area under the ROC curve (AUC) was used as a measurement to determine the correlation between survival and the expression of specific proteins. The cutoff point of expression for each protein that gave the maximum sum of sensitivity and specificity was used to divide the samples into a high-expression group and a low-expression group. Using the R package called ‘survival’, Kaplan-Meier curves were introduced to reveal the survival differences between samples in these groups. The Kaplan-Meier p-values (log-rank test) were also calculated with ‘survminer’ in R, which was used as another statistical value to evaluate the relationship between survival and the expression of specific proteins.

Literature background of the biomarkers

Of the three proteins highlighted by specific phosphosites, several links to melanoma were found in the literature. ADAM10, a member of a family of endopeptidases with broad specificity, is involved in the membrane shedding of several proteins. Interestingly, ADAM10 together with ADAM17 may promote membrane shedding of immunosuppressive proteins such as PDL1 and LAG3 [118–120]. FGA is a part of the glycoprotein fibrinogen, which has major functions in hemostasis, wound healing, and immune responses. High plasma levels of fibrinogen have been attributed to poor prognosis in lung cancer and melanoma. PTM state of fibrinogen has been linked to the disease state of cancer [154–158]. The third selected protein, HMOX1, is an antioxidant and anti-inflammatory enzyme involved in generating biliverdin and bilirubin. HMOX1 may promote cancer cell growth, tumor cell survival, and resistance to treatment. HMOX1 has earlier been linked to a poor outcome of melanoma [159–162].

Six other proteins were selected for IHC analysis based on regulation at the protein level:
SCAI is a highly conserved protein that acts on the RhoA–Dia1 pathway to regulate invasive cell migration. SCAI is downregulated in many human tumors, and high expression of SCAI correlates with better survival in patients with breast and lung cancers [135]; there is so far little information about SCAI in melanoma.

CDK4 is a well-known cancer target that regulates cell cycle and proliferation. In melanoma, mutations and dysregulation are commonly seen in CDK4 and proteins in its pathways, and several candidate drugs are in the clinical phase [125,126].

CTNND1 is a key regulator of cell-cell adhesion. Several studies suggest a link to melanoma. The long isoform (1A) was found regulated in our study. Longer isoforms, often enriched in tumors, have been reported as pro-tumorigenic, playing a role in EMT [163–166].

The helicase DDX11 has a role in chromatid cohesion. DDX11 has been reported upregulated with progression from noninvasive to invasive melanoma and expressed at high levels in advanced melanoma [136,138].

Glycodelin or PAEP, is a secreted glycoprotein that regulates critical steps during fertilization and has immunomodulatory effects. PAEP is expressed in melanoma and involved in tumor proliferation, and migration and may promote the development of immune tolerance in tumors. PAEP expression is regulated in part by MITF [167–171].

PIK3CB is part of the PI3K–AKT cascade, one of the most studied pathways in cancer. This pathway has a role in cell survival, migration, and oncogenic transformation. In melanoma, the PI3K–AKT pathway may be activated by mutations in NRAS or loss of suppressor PTEN [172–174].

Paired correlation analyses

To study the protein-based correlation between primary melanoma and metastasis, IHC analysis of the markers was also performed in nine additional metastases (Table S1B). First, a one-sample Kolmogorov-Smirnow test was conducted to determine the normality of the antibody expression data. For the comparison, the Wilcoxon signed-rank test was used to examine whether proteins were differentially expressed between the tumors and the matched metastases. P-values of the One-Sample Kolmogorov-Smirnow test and Wilcoxon signed-rank test were calculated by the IBM SPSS statistics package (26.0 version) software. $P < 0.05$ was considered statistically significant.

Survival analysis – Immunohistochemistry

To show the predictive impact of the nine markers, we performed independent t-tests to assess the differences in the means of the protein IHC expression values, quantified in the tumor cell and stroma parts, between the tumors of patients with OS of more than five years and less than five years. The assumption of homogeneity of variances was tested by Levene's Test of Equality of Variances. For the cases where significant differential expression was obtained, univariate

receiver operating characteristic (ROC) curve analysis was used to generate cutoff points for each protein that separated groups of patients with differences in OS. This was followed by Kaplan-Meier survival analyses based on models generated by the optimal cutpoint of each protein (Table S9A).

Independent t-test, ROC curve, Kaplan-Meier survival analysis, and figures including box plots showing p-values, quartile values, mean values, and 95% confidence intervals were produced by GraphPad IBM SPSS statistics package (version 26.0). $P < 0.05$ was considered statistically significant. Only samples from patients with OS > or <5 years (n=34) were included in the Mann-Whitney U t-test and ROC curve analyses.

Supplementary Figures

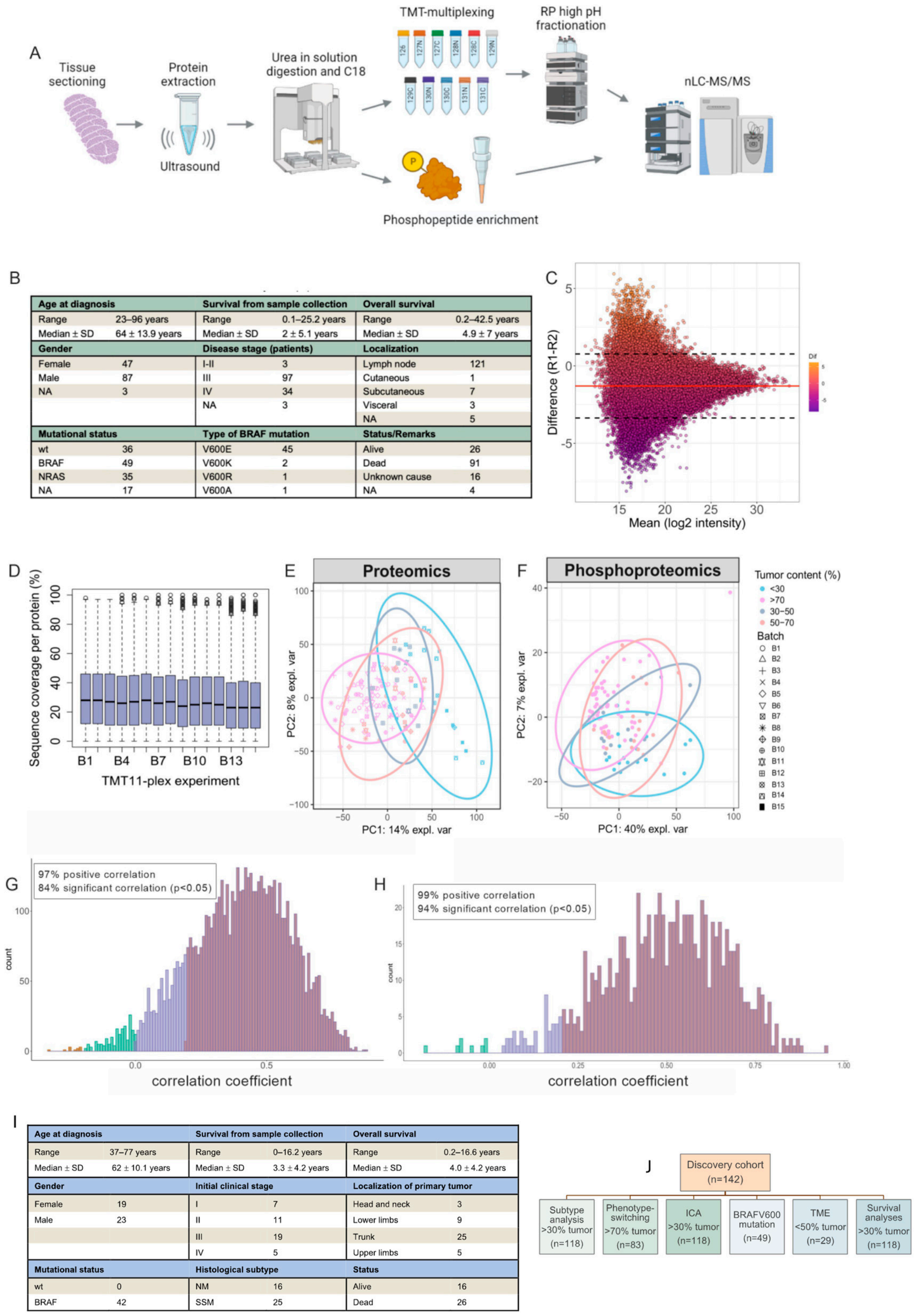

B

Age at diagnosis		Survival from sample collection		Overall survival	
Range	23–96 years	Range	0.1–25.2 years	Range	0.2–42.5 years
Median ± SD	64 ± 13.9 years	Median ± SD	2 ± 5.1 years	Median ± SD	4.9 ± 7 years
**Gender**		**Disease stage (patients)**		**Localization**	
Female	47	I-II	3	Lymph node	121
Male	87	III	97	Cutaneous	1
NA	3	IV	34	Subcutaneous	7
		NA	3	Visceral	3
				NA	5
**Mutational status**		**Type of BRAF mutation**		**Status/Remarks**	
wt	36	V600E	45	Alive	26
BRAF	49	V600K	2	Dead	91
NRAS	35	V600R	1	Unknown cause	16
NA	17	V600A	1	NA	4

I

Age at diagnosis		Survival from sample collection		Overall survival	
Range	37–77 years	Range	0–16.2 years	Range	0.2–16.6 years
Median ± SD	62 ± 10.1 years	Median ± SD	3.3 ± 4.2 years	Median ± SD	4.0 ± 4.2 years
**Gender**		**Initial clinical stage**		**Localization of primary tumor**	
Female	19	I	7	Head and neck	3
Male	23	II	11	Lower limbs	9
		III	19	Trunk	25
		IV	5	Upper limbs	5
**Mutational status**		**Histological subtype**		**Status**	
wt	0	NM	16	Alive	16
BRAF	42	SSM	25	Dead	26

Figure S1. Proteogenomics of melanoma metastases: workflow, cohort and quality control of the omics data
(A) Global Proteomic and phosphoproteomic workflow.

(B) Summary table of clinical features in the discovery cohort.
(C) Bland-Altman plot depicting the agreement across repeated experiments of batch one (B1), upper limit: 0.770, lower limit: -3.363, and mean difference: -1.297.
(D) Distribution of sequence coverage of the identified proteins by MS/MS across the fifteen TMT11 plex batches (whiskers show the 5–95 percentiles, and the dots represent the outliers).
(E) Principal component analysis of the TMT global proteome data after normalization and ratio calculation. The ellipses represent the 95% confidence interval per group based on 8,125 proteins.
(F) Principal component analysis of 1,267 commonly quantified phosphosites. The ellipses represent the 95% confidence interval per group.
(G) mRNA and protein abundance correlation (median 0.408), where 84% of the mRNA and protein pairs (6,101) showed a significant correlation (p-value 0.05) across 104 patient samples.
(H) Protein and phosphoprotein abundance correlation (median 0.506), where 94% of the proteins and phosphoprotein pairs (809) showed a significant correlation (p-value 0.05) across 94 patient samples.
(I) Summary table of clinical features in the cohort used for immunohistochemistry.
(J) Flowchart of the sample exclusion criteria for the different statistical analyses.

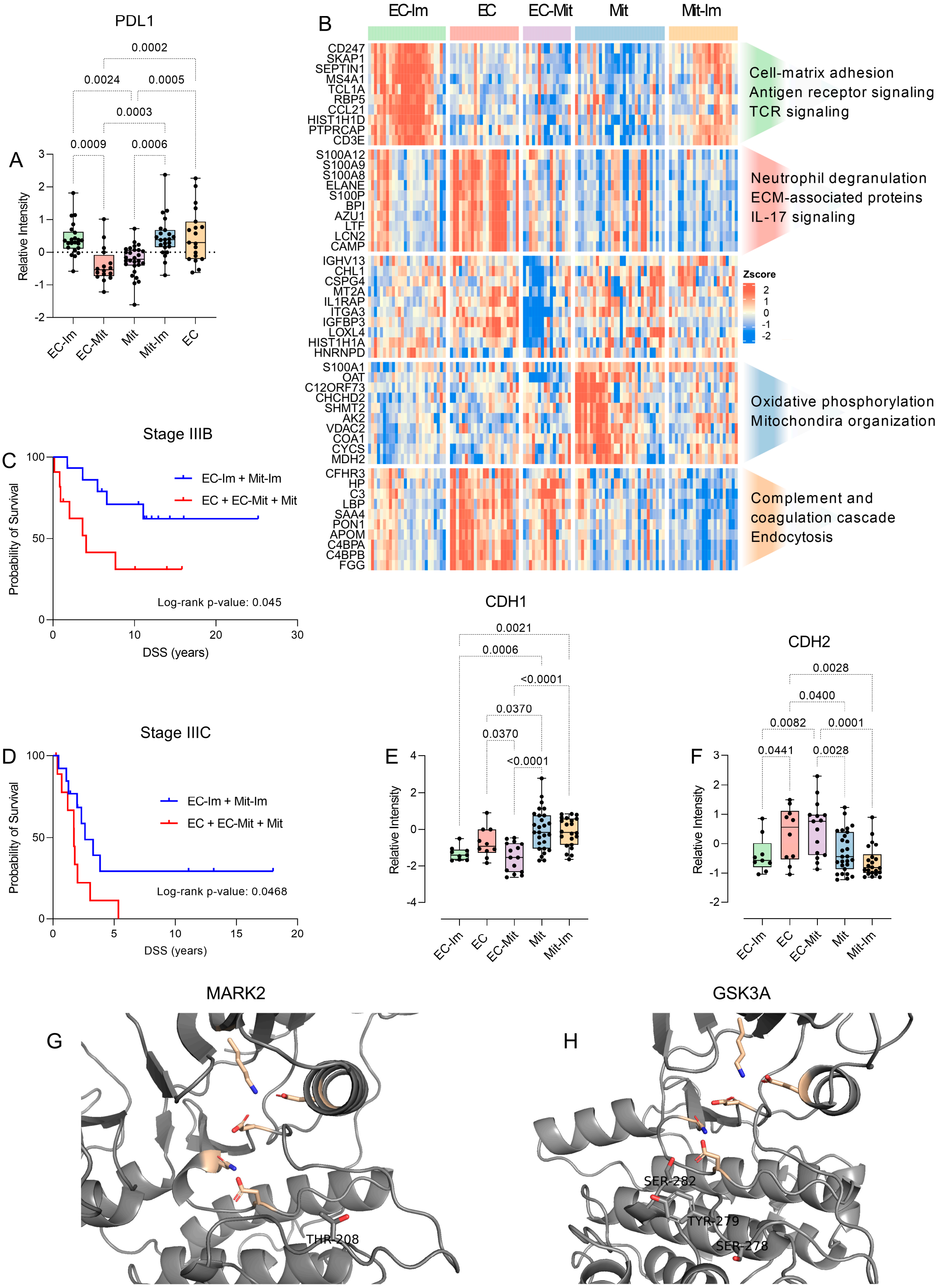

Figure S2. Molecular and clinical features of the proteomic subtypes

(A) PDL1 protein expression across the five proteomic subtypes.

(B) The ten proteins most strongly contributing to the ICs correlated with the proteomic subtypes and their enriched pathways (FDR < 0.05).

(C) Disease-specific survival probability for patients in disease stage IIIB, divided into subtypes associated with long and short survival.
(D) Disease-specific survival probability for patients in disease stage IIIC, divided into subtypes associated with long and short survival.
(E) Protein expression of CDH1 across the five proteomic subtypes.
(F) Protein expression of CDH2 across the five proteomic subtypes.
(G) Location of regulated phosphosites in the activation loops within three-dimensional structures of the MARK2 kinase.
(H) Location of regulated phosphosites in the activation loops within three-dimensional structures of the GSK3A kinases.

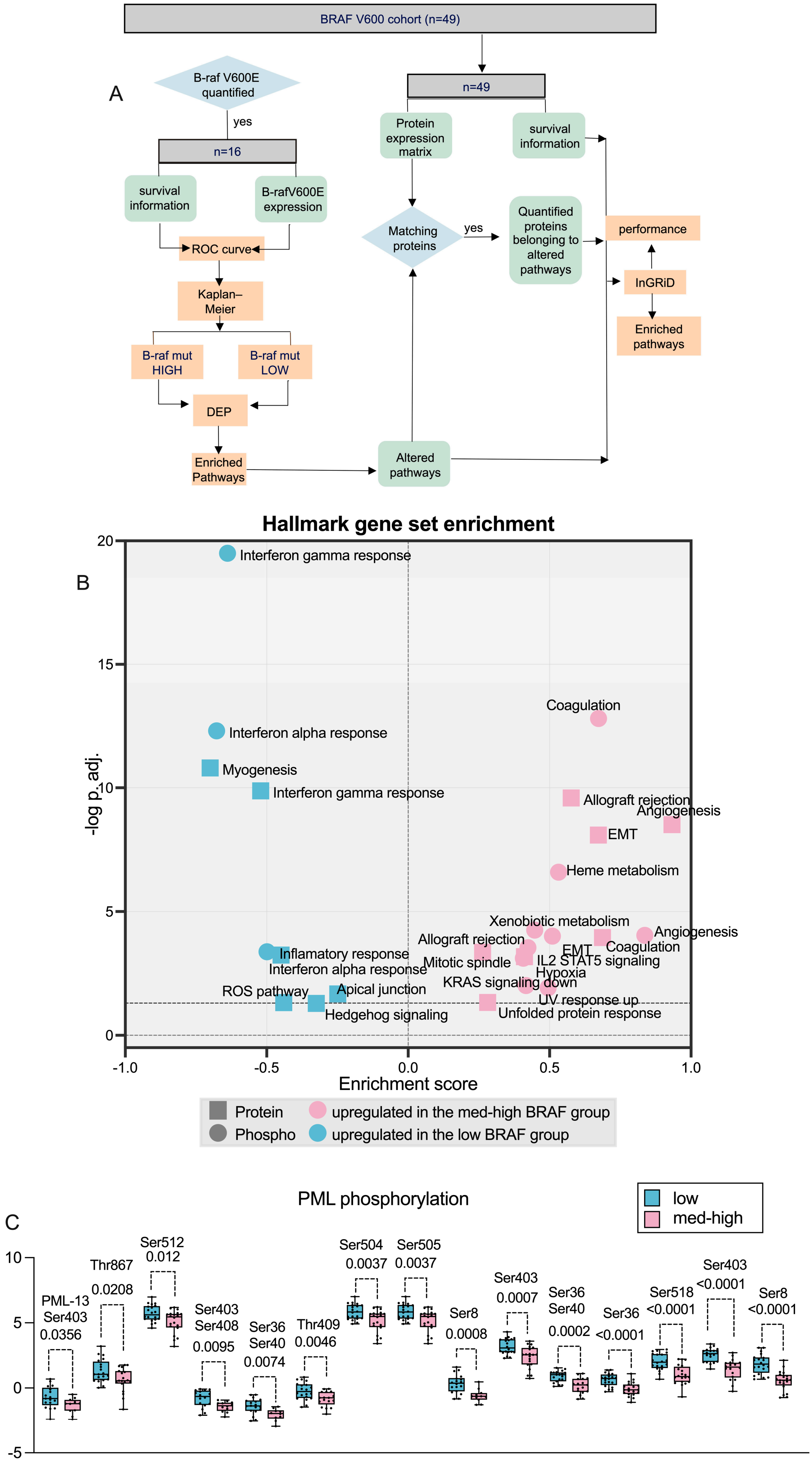
A
BRAF V600 cohort (n=49)
B-raf V600E quantified
yes
n=16
survival information
B-rafV600E expression
ROC curve
Kaplan–Meier
B-raf mut HIGH
B-raf mut LOW
DEP
Enriched Pathways
n=49
Protein expression matrix
survival information
Matching proteins
yes
Quantified proteins belonging to altered pathways
performance
InGRiD
Enriched pathways
Altered pathway
B
Hallmark gene set enrichment
Interferon gamma response
Coagulation
Interferon alpha response
Myogenesis
Interferon gamma response
Allograft rejection
Angiogenesis
EMT
Heme metabolism
Xenobiotic metabolism
Angiogenesis
Allograft rejection
EMT
Coagulation
Inflamatory response
Mitotic spindle
IL2 STAT5 signaling
Interferon alpha response
Hypoxia
KRAS signaling down
ROS pathway
Apical junction
UV response up
Hedgehog signaling
Unfolded protein response
-log p.adj.
Enrichment score
Protein
Phospho
upregulated in the med-high BRAF group
upregulated in the low BRAF group
C
PML phosphorylation
low
med-high
PML-13 Ser403 0.0356
Thr867 0.0208
Ser512 0.012
Ser403 Ser408 0.0095
Ser36 Ser40 0.0074
Thr409 0.0046
Ser504 0.0037
Ser505 0.0037
Ser8 0.0008
Ser403 0.0007
Ser36 Ser40 0.0002
Ser36 <0.0001
Ser518 <0.0001
Ser403 <0.0001
Ser8 <0.0001

Figure S3. Molecular difference between the low and med-high BRAF V600 mortality risk groups

(A) Workflow for the association between BRAF V600E expression and patient survival in a larger cohort. The orange squares indicate statistical tests.

(B)Enrichment of the differentially expressed proteins (p-value 0.05) and phosphosites between the low and medium-high mortality risk groups using the Hallmark gene set.

(C) Phosphosite expression of the PML protein and the PML-13 isoform between the low and medium-high mortality risk groups.

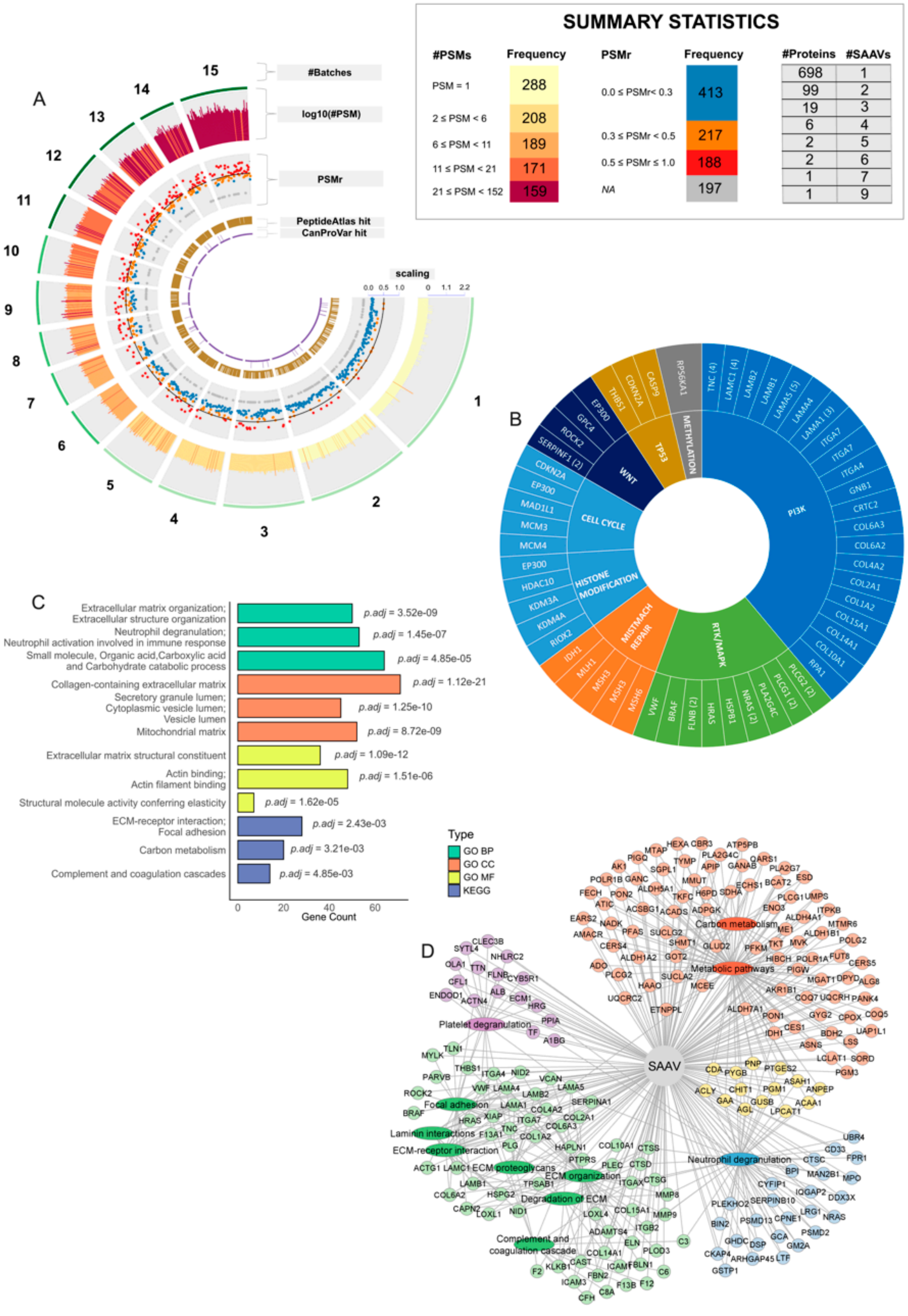

Figure S4. Overview of identified SAAVs

(A) Summary of the 1,015 validated SAAVs, including the number of TMT batches, number of PSMs, SAAVf, and the availability in PeptideAtlas and CanProVar databases. The embedded

summary statistics table includes bar charts showing the number of PSMs (#PSMs), the SAAVf, and the distribution of the number of SAAVs per protein.
(B) Identified Genes with SAAVs belonging to signaling pathways frequently dysregulated in melanoma.
(C) KEGG and GO enrichment analysis of the 828 proteins with SAAVs.
(D) Network of proteins with SAAVs linked to KEGG and GO-enriched pathways.

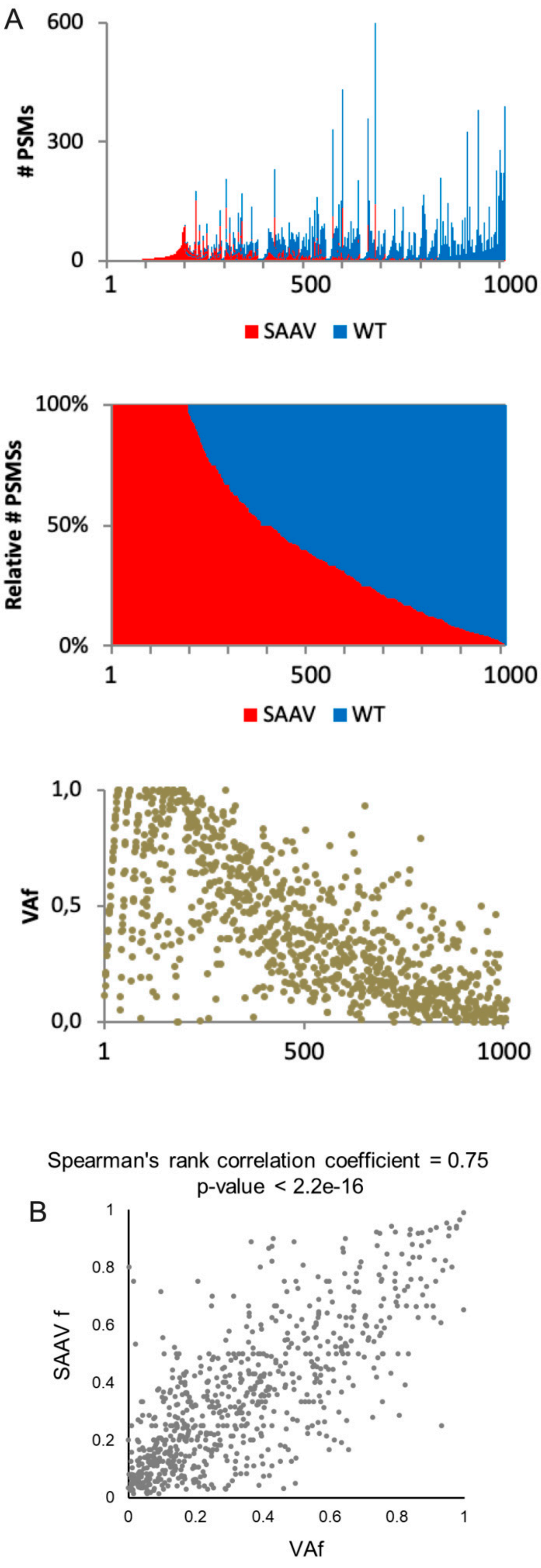
A
600
300
0
PSMs
1
500
1000
SAAV
WT
100%
50%
0%
Relative # PSMSs
1
500
1000
SAAV
WT
1,0
0,5
0,0
VAf
1
500
1000
Spearman's rank correlation coefficient = 0.75
p-value < 2.2e-16
B
1
0.8
0.6
0.4
0.2
0
SAAV f
0
0.2
0.4
0.6
0.8
1
VAf

Figure S5. Protein expression of variants and WT counterparts.

(A)In the y-axis, #PSM for the wt and SAAV (top panel), ratio #PSMs-wt/#PSMs-SAAV (middle panel), and SAAV variant allele frequencies (bottom panel). The SAAVs were ranked (x-axis) according to the percentage of PSMs of wt counterparts from the total number of PSMs, including SAAV and wt.

(B) Significant correlation between SAAVf and VAf.

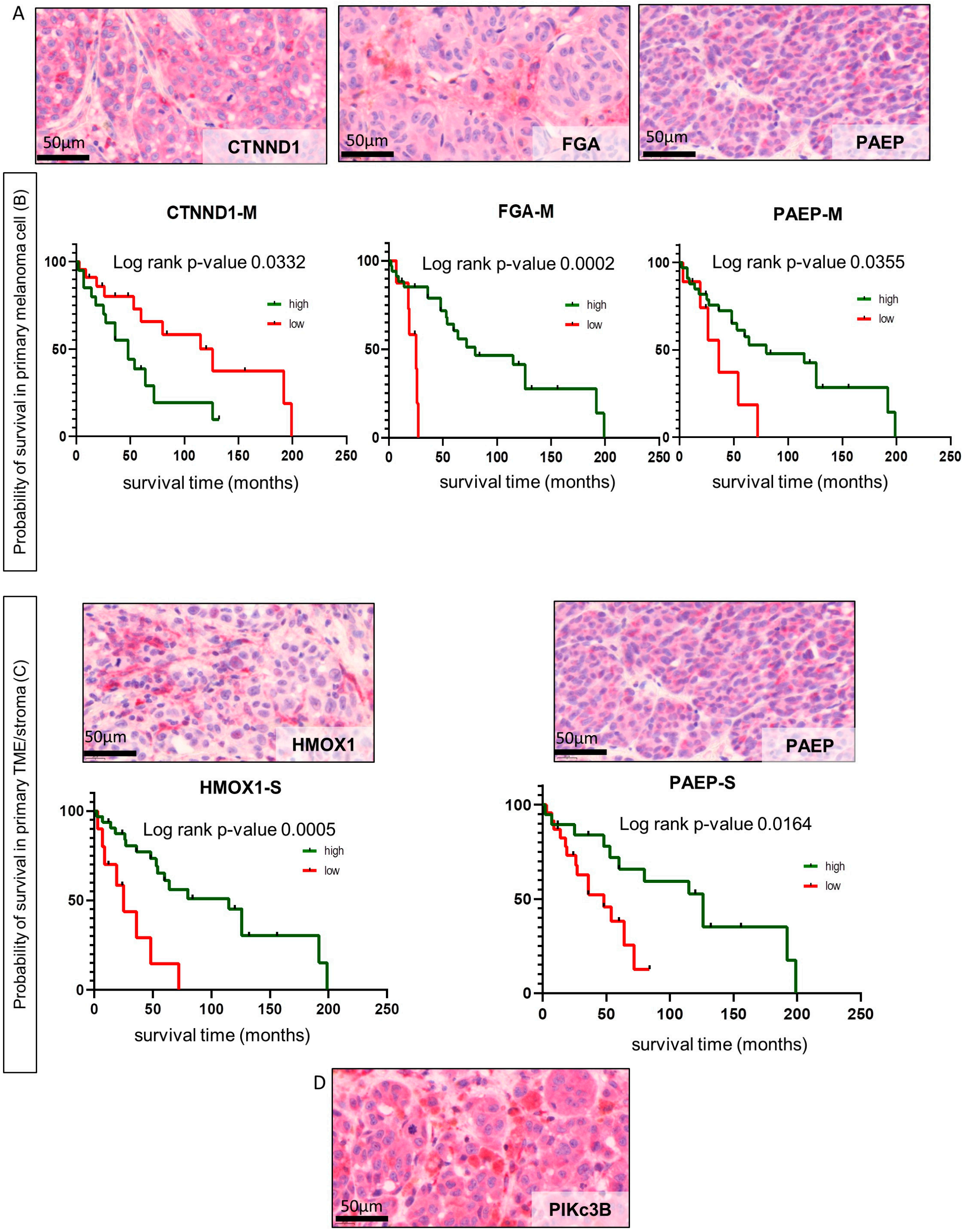

Figure S6. Different expressional features of the candidate biomarkers and survival during progression

A) Tissue heterogeneity of CTNND1, FGA, and PAEP protein expression in melanoma tissue. IHC, fast red colorimetry – OM 112x; scale bar 50µm.

B) Kaplan-Meier analyses of the OS rates for patients in association with high (green) and low (red) expression of the markers (CTNND1, PAEP, FGA) in melanoma cells with significant differences.

C) IHC expression and Kaplan-Meier analyses of the OS rates for patients with high (green) and low (red) expression of HMOX1 and PAEP in the TME with significant differences. IHC, fast red colorimetry – OM 112x; scale bar 50µm.
D) Tissue heterogeneity of PIKc3B protein IHC expression.

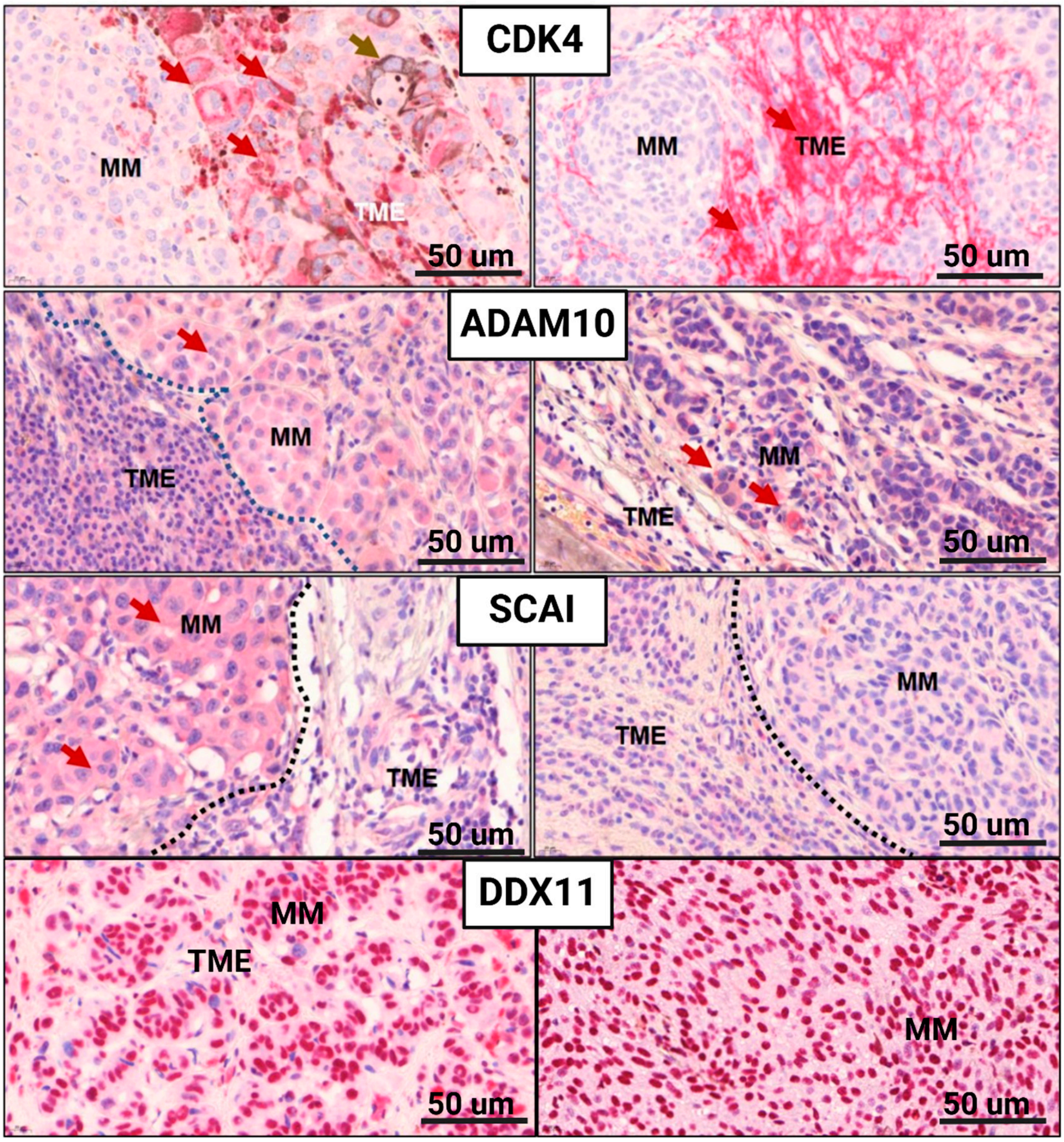

Figure S7. Representative IHC stainings for all four proteins. The figure illustrates the IHC staining distribution within the tumor and the surrounding stroma. (OM - optimal magnitude - 112×). Red arrows indicate the tumor microenvironment (TME), highlighting the stromal microenvironment of the melanoma, which consists of fibroblasts and inflammatory cells embedded within the extracellular matrix. The melanoma cells (MM) represent the tumoral cell compartment of the melanoma tissue, denoted by brown arrows.
